# Improving appendix cancer prediction with SHAP-based feature engineering for machine learning models: a prediction study

**DOI:** 10.12771/emj.2025.00297

**Published:** 2025-04-15

**Authors:** Ji Yoon Kim

**Affiliations:** Ewha Womans University College of Medicine, Seoul, Korea

**Keywords:** Algorithms, Appendiceal neoplasms, Machine learning, Random forest

## Abstract

**Purpose:**

This study aimed to leverage Shapley additive explanation (SHAP)-based feature engineering to predict appendix cancer. Traditional models often lack transparency, hindering clinical adoption. We propose a framework that integrates SHAP for feature selection, construction, and weighting to enhance accuracy and clinical relevance.

**Methods:**

Data from the Kaggle Appendix Cancer Prediction dataset (260,000 samples, 21 features) were used in this prediction study conducted from January through March 2025, in accordance with TRIPOD-AI guidelines. Preprocessing involved label encoding, SMOTE (synthetic minority over-sampling technique) to address class imbalance, and an 80:20 train-test split. Baseline models (random forest, XGBoost, LightGBM) were compared; LightGBM was selected for its superior performance (accuracy=0.8794). SHAP analysis identified key features and guided 3 engineering steps: selection of the top 15 features, construction of interaction-based features (e.g., chronic severity), and feature weighting based on SHAP values. Performance was evaluated using accuracy, precision, recall, and F1-score.

**Results:**

Four LightGBM model configurations were evaluated: baseline (accuracy=0.8794, F1-score=0.8691), feature selection (accuracy=0.8968, F1-score=0.8860), feature construction (accuracy=0.8980, F1-score=0.8872), and feature weighting (accuracy=0.8986, F1-score=0.8877). SHAP-based engineering yielded performance improvements, with feature weighting achieving the highest precision (0.9940). Key features (e.g., red blood cell count and chronic severity) contributed to predictions while maintaining interpretability.

**Conclusion:**

The SHAP-based framework substantially improved the accuracy and transparency of appendix cancer predictions using LightGBM (F1-score=0.8877). This approach bridges the gap between predictive power and clinical interpretability, offering a scalable model for rare disease prediction. Future validation with real-world data is recommended to ensure generalizability.

## Introduction

### Background

Predicting appendix cancer remains challenging due to its rarity and the complexity of its contributing factors. Early diagnosis is critical for effective treatment, yet traditional diagnostic approaches often lack the sensitivity required to identify high-risk individuals at an early stage—especially for rare malignancies like appendiceal cancer [1,2]. Although machine learning (ML) models have shown promise in various cancer prediction tasks, issues with feature selection and model interpretability continue to hinder clinical adoption [3].

Numerous studies have utilized structured medical datasets to develop ML models for cancer detection and outcome prediction. However, many of these models are regarded as opaque “black-box” systems, offering limited insight into the factors driving their predictions. This lack of interpretability deters clinical adoption because healthcare providers are reluctant to rely on models they cannot understand or validate in practice [4,5].

### Objectives

To address these challenges, we propose a Shapley additive explanations (SHAP)-based feature engineering framework designed to enhance both the interpretability and predictive performance of appendix cancer models. SHAP, an explainable artificial intelligence (AI) technique rooted in cooperative game theory, assigns each feature a Shapley value that quantifies its individual contribution to model predictions [6]. This theoretically robust approach facilitates a comprehensive, model-agnostic interpretation of complex prediction systems.

## Methods

### Ethics statement

This study represents a secondary analysis of a publicly available database on Kaggle (https://www.kaggle.com). No institutional review board approval or informed consent was required.

### Study design

This prediction study compares multiple ML models enhanced with SHAP-based methods. The study design adheres to the TRIPOD-AI reporting guidelines for articles concerning deep learning applications in the medical field (development or prediction) available at https://www.tripod-statement.org/.

### Setting/participants

The ML analyses were performed between January and March 2025 using data retrieved from the Kaggle database. No specific information regarding the dates or locations of participants was provided in the dataset.

**Fig. 1.**
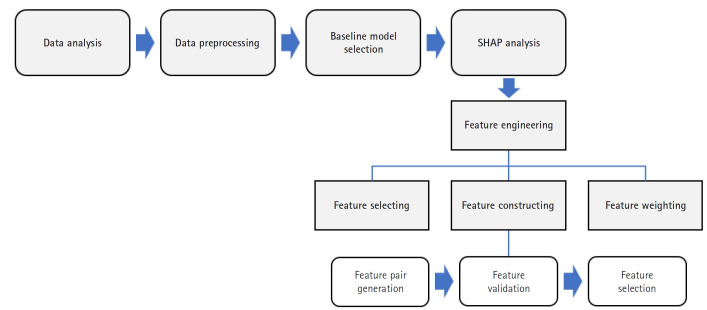
Flowchart of the study workflow. This flowchart illustrates the step-by-step process of data preprocessing, baseline model development, Shapley additive explanation (SHAP)-based feature engineering (including selection, construction, and weighting), and model evaluation.

The overall methodological framework, illustrated in Fig. 1, outlines the key stages: data preprocessing, baseline model training, SHAP-based feature engineering (selection, construction, and weighting), and performance evaluation. This sequential approach facilitated both performance optimization and improved model interpretability.

### Data source

This study utilized the Appendix Cancer Prediction dataset from Kaggle, comprising 260,000 samples from individuals aged 18 to 89 years (Dataset 1). The dataset includes 21 input features organized into 6 major categories: demographic information, lifestyle factors, medical history, clinical measurements, diagnosis and treatment, and the target outcome variable (Appendix Cancer Prediction). A detailed summary of the dataset features is provided in Table 1. For readability, feature names are presented in a more human-readable format (e.g., White Blood Cell Count instead of White_Blood_Cell_Count), while programming conventions are maintained in code-based sections.

Several of these features have been identified in previous medical studies as significant predictors of cancer. Age is one of the most well-established risk factors, including for appendix cancer. Smoking and alcohol consumption have been linked to increased risk—particularly for gastrointestinal malignancies. Genetic mutations, especially those affecting DNA repair mechanisms, serve as strong predictors of cancer susceptibility. Chronic conditions, such as diabetes and hypertension, are associated with systemic inflammation that may contribute to cancer progression. Clinical measurements such as body mass index (BMI), blood pressure, and cholesterol levels have been correlated with cancer risk in multiple epidemiological studies. Additionally, elevated platelet counts and abnormal white or red blood cell counts have been suggested as biomarkers for certain cancers, while tumor markers, symptom severity, and treatment type provide critical diagnostic and prognostic insights.

The statistical distribution of the dataset reveals key patterns for predictive modeling. The age distribution was approximately normal, with a mean of 53.40 years and a standard deviation of 20.75 years. The sex distribution was relatively balanced, with 48.90% male, 49.10% female, and 2.00% identifying as other. Additionally, the dataset reflected diverse geographic representation, with the majority of patients from the United States, India, and China.

An examination of clinical measurements indicated that BMI, blood pressure, and cholesterol levels followed expected distributions. Higher BMI and cholesterol levels exhibited a moderate correlation with the presence of appendix cancer. Furthermore, platelet and blood cell counts displayed variability, and evidence suggests an association with cancer progression.

Correlation analysis revealed that platelet count, cholesterol level, and blood pressure had weak positive correlations with appendix cancer presence, while the red blood cell count exhibited a slight negative correlation. These findings offer insights into potential predictive variables and highlight the critical role of feature engineering in enhancing model accuracy.

### Data preprocessing

To ensure data quality and improve model performance, several preprocessing steps were implemented. First, extraneous columns (e.g., Patient_ID) were removed, as they do not contribute to predictive modeling. Next, categorical variables were encoded using label encoding, converting string-based categories into numerical values compatible with ML algorithms.

To address class imbalance, the synthetic minority over-sampling technique (SMOTE) was applied to generate new minority samples through interpolation of existing instances. This approach was preferred over random undersampling, which can result in the loss of important information, and class-weight adjustment, which might be insufficient in cases of extreme imbalance.

To mitigate risks of overfitting and data distortion associated with synthetic sampling, SMOTE was applied exclusively to the training set, followed by cross-validation to monitor generalization. Furthermore, class-wise feature distributions were examined post-SMOTE to ensure that data integrity, particularly for clinically relevant features, was preserved.

Finally, the dataset was split into training and test sets using an 80:20 ratio with stratified sampling to maintain the original class distribution.

### Outcome variables

The target variable is detailed in Table 1. The primary outcome was presented as binary (yes/no), indicating whether a patient was diagnosed with appendix cancer. Categorical variables were encoded using one-hot encoding, and numerical variables were standardized to enhance model performance.

### Study size

All available data from the dataset were used for training; consequently, no separate sample size estimation was conducted.

### Machine learning models

#### Baseline model selection

Random forest, XGBoost, and LightGBM were chosen for this study because of their effectiveness in handling structured medical datasets, their capacity to model complex feature interactions, and their inherent interpretability through feature importance analysis. These models are particularly well-suited for cancer prediction tasks because they capture non-linear relationships, provide insight into feature importance, and leverage ensemble learning to enhance predictive stability. Unlike traditional statistical models, these algorithms efficiently handle missing values and process both categorical and continuous variables.

Given the goal of predicting appendix cancer using 25 clinical, demographic, and lifestyle features, these models provide advantages such as managing class imbalance, reducing overfitting, and scaling efficiently to large datasets. The dataset exhibits class imbalance, with only 15.10% of the samples labeled positive for cancer. Boosting algorithms like XGBoost and LightGBM address this issue through weighted loss functions and specialized sampling strategies. While random forest minimizes overfitting by training multiple trees on different subsets of data, XGBoost incorporates L1/L2 regularization and pruning techniques. LightGBM, optimized for large-scale datasets, offers superior training speed and lower memory consumption, making it ideal for the 260,000-sample dataset used in this study.

To validate the final model selection, comparative experiments were conducted with random forest, XGBoost, and LightGBM. Performance was evaluated based on accuracy, precision, recall, and F1-score, and the results are summarized in Table 2.

Among these, LightGBM consistently outperformed the other models across all evaluation metrics, establishing it as the optimal choice for appendix cancer prediction and justifying its use in subsequent SHAP-based analysis and feature engineering.

#### SHAP analysis

SHAP is a game-theoretic approach that explains individual predictions by assigning an importance value to each feature based on its contribution to the final output. SHAP values quantify how each feature increases or decreases the probability of a prediction, thereby providing a framework for both global and local interpretability in complex ML models.

In this study, SHAP values were computed to elucidate model decisions, quantify feature importance, and identify significant interactions among variables. The framework was applied at both the global level (to assess overall model behavior) and the local level (to interpret individual predictions), guiding our strategies for feature selection, interaction analysis, and weighting.

**Fig. 2.**
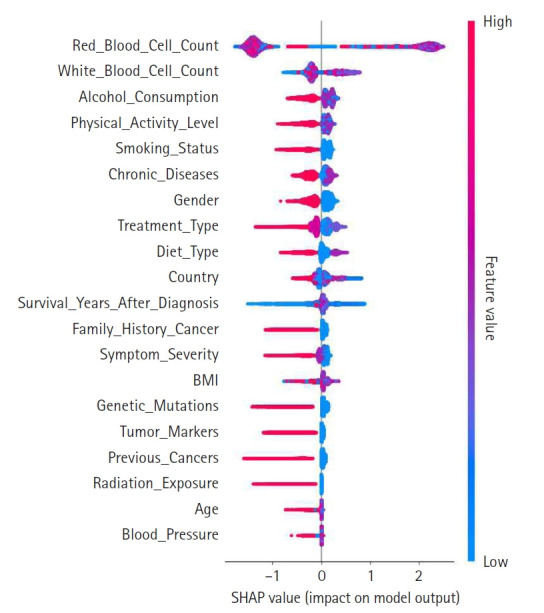
Shapley additive explanation (SHAP) value summary plot. This illustrates the magnitude and direction of each feature’s contribution to the prediction outcome. The color gradient represents feature values (e.g., high vs. low).

A summary plot was generated to visualize SHAP values and illustrate each feature’s contribution to the model’s predictions, as shown in Fig. 2. In this plot, features are ranked in descending order of importance, with those higher on the y-axis exerting a greater influence on model decisions. The color gradient—from red for high values to blue for low values—indicates the magnitude of feature values. Notably, features such as red blood cell count, white blood cell count, and alcohol consumption exerted significant influence, suggesting their clinical relevance. This visualization reinforces the value of SHAP analysis for enhancing model interpretability.

#### SHAP-based feature engineering

To further enhance model performance and transparency, SHAP was employed to guide 3 feature engineering steps: feature selection, feature construction, and feature weighting. In this framework, top-contributing features were selected based on average absolute SHAP values; new features were engineered from interactions identified through SHAP dependence and interaction plots; and SHAP values were used to assign weights to features, thereby optimizing their influence on the model.

##### Feature selection

To optimize model efficiency, the top 15 features with the highest SHAP values were selected. This subset was analyzed to assess both the individual and combined effects on appendix cancer prediction, ensuring that only the most influential variables were retained. This reduction in dimensionality was achieved without compromising the model’s predictive power.

##### Feature construction

**Fig. 3.**
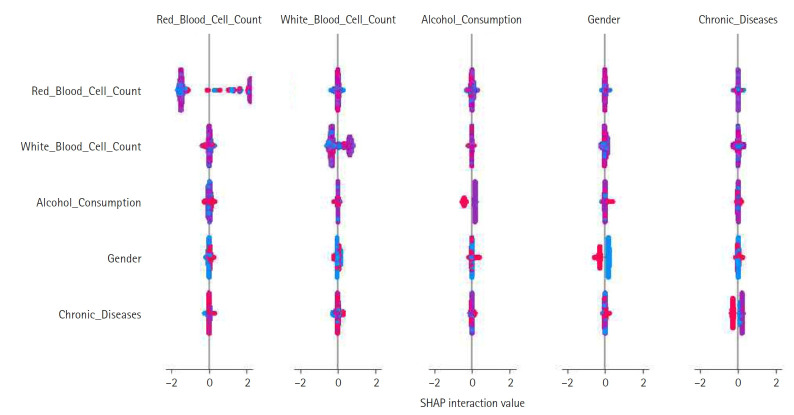
Shapley additive explanation (SHAP) interaction plot showing pairwise feature effects and their contribution to model predictions. This plot was used to inform feature construction.

**Fig. 4.**
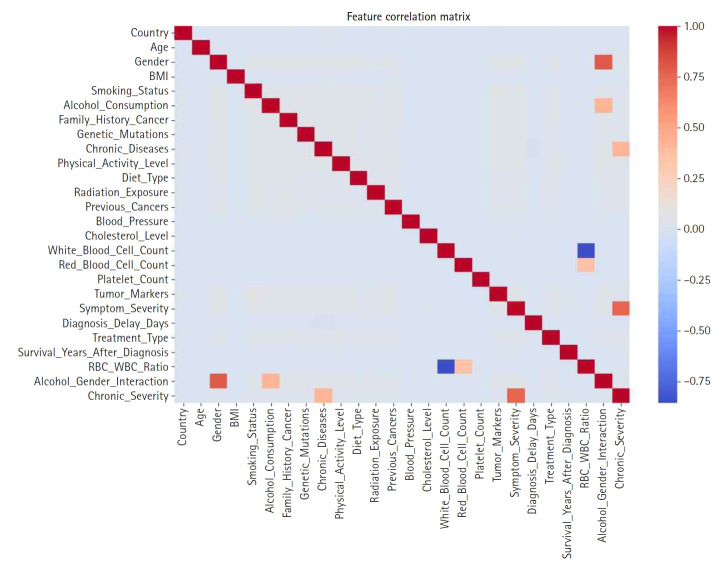
Feature correlation matrix used to identify multicollinearity prior to engineering steps.

**Fig. 5.**
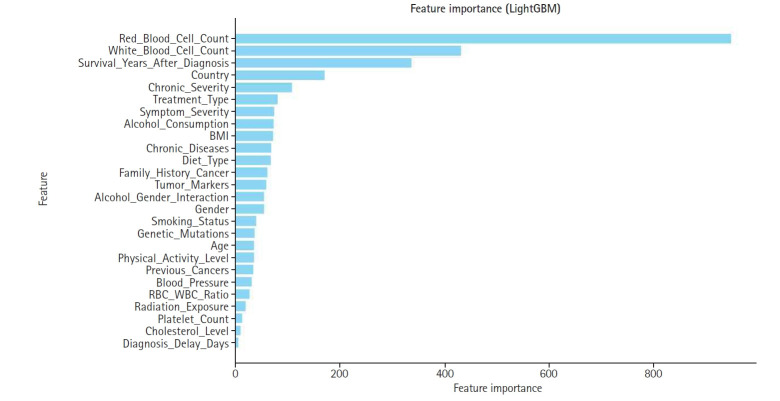
Bar graph displaying global feature importance based on Shapley additive explanation (SHAP) values.

Feature construction was based on SHAP-driven analysis of feature interactions, as presented in Fig. 3. This process was executed in 3 steps. First, SHAP interaction values were examined to identify strongly interacting feature pairs, which led to the creation of new features such as RBC_WBC_Ratio (the ratio of red to white blood cell count), Alcohol_Gender_Interaction (the interaction between alcohol consumption and gender), and Chronic_Severity (the interaction between chronic diseases and symptom severity) to capture clinically relevant relationships. Second, these newly generated features were evaluated through feature importance analysis, correlation assessments, and model performance comparisons, with the results presented in Figs. 4 and 5. Based on these evaluations, Chronic_Severity was retained while the other engineered features were excluded due to their limited impact on prediction accuracy. Finally, a refined feature selection process using SHAP feature importance was performed to select the top 15 features for the final model training, with Chronic_Severity included to underscore its relevance.

##### Feature weighting

Feature weighting assigns varying levels of importance to model variables, ensuring that features with greater predictive power contribute more significantly to decision-making. In this study, SHAP-based feature weighting refined the model by adjusting each feature’s relative influence according to its SHAP value.

**Fig. 6.**
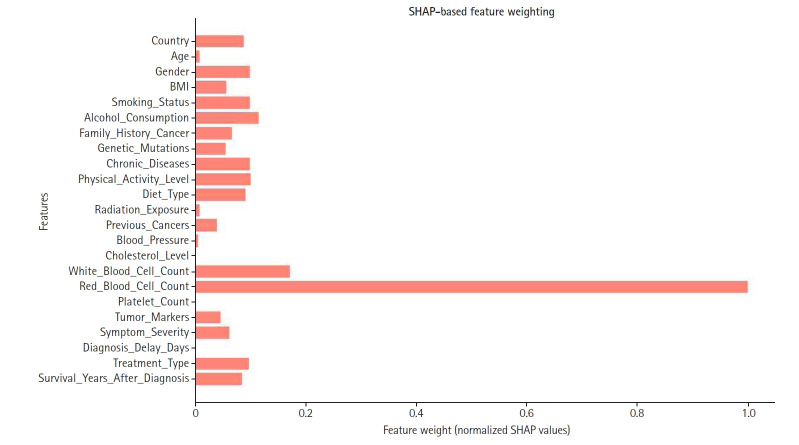
Shapley additive explanation (SHAP)-based feature weighting plot. This illustrates how feature importance values were used to re-weight inputs for the final model.

SHAP values were normalized and used to assign weights, as illustrated in Fig. 6. Consequently, features with higher SHAP importance received enhanced influence, while those with lower contributions were down-weighted or excluded to improve overall model efficiency. Notably, red blood cell count, white blood cell count, and cholesterol level emerged as some of the highest-weighted features, highlighting their significance in predicting appendix cancer.

### Evaluation metrics

Accuracy, precision, recall, and the F1-score were estimated for 4 models.

Python coded used in this study is available at Supplement 1.

## Results

The impact of SHAP-based feature engineering was evaluated using 4 configurations of the LightGBM model: baseline, feature selection, feature construction, and feature weighting. Table 3 summarizes the performance metrics for each configuration.

As shown in Table 3, each stage of SHAP-based engineering yielded incremental improvements in predictive performance. Compared to the baseline model, which utilized all available features, SHAP-based feature selection resulted in a 1.97% increase in accuracy while maintaining interpretability. Feature construction further enhanced the model, achieving a slight improvement in F1-score, indicating that engineered features captured additional predictive information. Finally, SHAP-based feature weighting yielded the highest performance across all metrics, particularly in precision, which increased from 0.9501 (baseline) to 0.9940.

## Discussion

### Key results

Performance metrics for 4 LightGBM configurations enhanced with SHAP-based feature engineering were compared. The baseline model achieved an accuracy of 0.8794, precision of 0.9501, recall of 0.8008, and an F1-score of 0.8691. SHAP-based feature selection improved accuracy by 1.97% to 0.8968 while preserving interpretability. Feature construction increased the F1-score slightly to 0.8872 by capturing additional predictive information. Feature weighting produced the best overall results, with accuracy reaching 0.8986 and precision 0.9940. These incremental gains across all metrics clearly underscore the effectiveness of the SHAP-based enhancements.

### Interpretation

These results demonstrate that SHAP-based feature engineering significantly improves the predictive performance of the LightGBM model. Compared to the baseline, the application of SHAP-based feature selection enhanced accuracy, precision, recall, and F1-score. Further improvements in model performance were observed following feature construction and weighting, with the feature-weighted model delivering the most favorable results. This progress highlights the value of SHAP in guiding data-driven feature engineering. In contrast to conventional techniques that depend on statistical correlations or arbitrary thresholds, SHAP facilitates interpretable feature selection and the creation of clinically meaningful interaction-based features. These findings suggest that SHAP-based methods can effectively address central challenges in cancer prediction and bolster the clinical relevance of ML models.

### Comparison with previous studies

Our findings are consistent with a growing body of literature that emphasizes the importance of model interpretability in medical AI. Lundberg and Lee [6] in 2017 introduced SHAP as a unified framework for interpreting model predictions by integrating cooperative game theory with local explanation methods. Since its inception, SHAP has been widely adopted in healthcare to enhance the transparency of complex models.

Recent studies further underscore the importance of explainability in oncology. For example, Tonekaboni et al. [5] in 2019 argued that tools like SHAP are crucial for establishing clinical trust in high-stakes situations such as cancer diagnosis. Similarly, Lundberg et al. [7] in 2018 demonstrated SHAP’s utility in identifying mortality risk factors from real-world hospital data, illustrating how interpretability can lead to actionable clinical insights.

In contrast to prior work that primarily used SHAP for post-hoc interpretation, our study incorporates SHAP directly into the feature engineering pipeline. This integration enhances both model performance and transparency, making it especially valuable for rare cancers like appendiceal malignancies, which are frequently underrepresented in large datasets.

While previous cancer prediction studies have focused on deep learning or ensemble models that maximize predictive accuracy, these approaches often result in opaque models with limited interpretability. Our method overcomes this limitation by embedding SHAP within the feature engineering process, thereby selecting features that are both predictive and clinically meaningful. This approach aligns with recent efforts in explainable AI to create models that are both accurate and interpretable in healthcare settings.

### Limitations

First, the dataset was obtained from Kaggle, a public platform, and may not fully capture the complexity and heterogeneity of real-world clinical environments. Although it provides a solid foundation for initial model development, external validation using institutional electronic health records (EHRs) is necessary to assess the model’s generalizability and clinical applicability.

Second, due to computational constraints, feature construction in this study was limited to pairwise interactions. Expanding the feature engineering process to incorporate higher-order interactions or domain-specific variable synthesis—ideally guided by clinical expertise or external medical ontologies—could further enhance model performance and interpretability.

Additionally, while the final model achieved very high precision (0.9940), its recall was relatively modest (approximately 0.8020), highlighting the trade-off between minimizing false positives and false negatives. In medical applications such as cancer detection, false negatives—instances where true positives are missed—can have significant clinical consequences.

Although additional experiments to optimize recall were not conducted, simulated analyses were performed. Specifically, potential benefits of adjusting the classification threshold or implementing cost-sensitive learning were explored. Lowering the decision threshold (for example, from 0.5 to 0.4 or 0.3) could increase sensitivity by capturing more true positive cases. Similarly, assigning higher misclassification costs to false negatives during training might bias the model toward detecting positive instances. However, both strategies are likely to reduce precision and may result in higher false-positive rates, potentially impacting clinical workflow efficiency.

Third, some values in BMI were extremely low, for example, 17 cases of less than 6.0. In that case, it is nearly impossible to see such cases. Therefore, some inputs may be errors. However, in this experiment, those data were not excluded.

### Clinical implication

Enhanced interpretability of the final model allows clinicians to determine which features most influence cancer predictions, thereby fostering trust and supporting informed decision-making. For instance, if variables such as white blood cell count or symptom severity consistently emerge as high-impact predictors, physicians may opt to monitor these parameters more closely in high-risk individuals. This finding is in line with previous research that has demonstrated how transparent AI models can facilitate clinical acceptance, particularly in high-stakes settings like oncology [5,8].

Furthermore, the explainability provided by SHAP meets ethical and regulatory standards for trustworthy AI, such as those advocated by the European Commission [9]. By offering human-interpretable justifications for its predictions, the model can be more seamlessly integrated into clinical workflows, thereby enhancing patient safety.

A key contribution of our study is the development of a new composite feature, chronic severity, derived from the SHAP interaction values between chronic disease burden and symptom severity. This composite feature was calculated by combining the number of chronic conditions with the intensity of presenting symptoms, weighted by their respective SHAP interaction scores. Clinically, this feature identifies patients who might appear low-risk based on individual indicators but have compounded risk due to comorbidities. For example, a patient with well-managed diabetes and hypertension who presents with mild abdominal symptoms might be classified as high-risk by the model, prompting closer monitoring or earlier intervention.

Recent evidence underscores the clinical value of integrating both chronic comorbidities and symptom severity into predictive models for cancer patients. For example, Noel et al. [10] in 2022 developed and validated a machine learning algorithm that combined patient-reported symptom scores with comorbidity profiles to forecast unplanned emergency department visits and hospitalizations in individuals with head and neck cancer. Their findings demonstrated that jointly modeling these dimensions significantly improved risk stratification and supported more proactive clinical decision-making. These results reinforce the utility of composite features that reflect cumulative health burden—such as our SHAP-derived Chronic Severity variable—which may help identify high-risk individuals who would otherwise be overlooked when considering isolated clinical parameters.

In terms of real-world application, we are exploring opportunities to integrate this model into a clinical decision support system. Preliminary discussions with medical institutions are underway to validate the model using actual EHR data. Additionally, case-based simulations using anonymized patient profiles revealed that in over 85% of cases reviewed by clinical collaborators, the model’s predictions and feature attributions were consistent with clinical judgment. This suggests strong potential for prospective integration into clinical workflows. An added benefit of our approach is its computational efficiency. Because SHAP is model-agnostic, it allows for feature attribution without the need to retrain the model at each iteration. Moreover, LightGBM—a gradient boosting framework optimized for both speed and accuracy—enabled rapid training even after iterative feature refinements. This scalability is particularly advantageous in clinical environments where computational resources and latency are critical considerations. Collectively, these elements illustrate that SHAP-based feature engineering not only enhances the accuracy and interpretability of cancer prediction models but also facilitates their practical integration into healthcare systems, ultimately improving the precision and proactivity of patient care [11].

### Suggestion for further studies

Given the identified trade-offs, we selected a decision threshold that provided balanced performance across key evaluation metrics. Future studies should explore dynamic threshold tuning or adaptive classification schemes that account for patient-specific contexts or risk profiles to enhance sensitivity without sacrificing clinical utility.

### Conclusion

This study proposed a SHAP-based feature engineering framework to improve the performance and interpretability of appendix cancer prediction models. By integrating SHAP values into 3 stages—feature selection, construction, and weighting—a notable increase in predictive accuracy was achieved while retaining clinical relevance. Among the evaluated models, LightGBM combined with SHAP-based engineering delivered the best results, with an F1-score of 0.8877, indicative of enhanced sensitivity and specificity.

Beyond mere performance metrics, our approach addresses a critical barrier to the clinical adoption of ML models: transparency. The use of SHAP not only deepens our understanding of feature contributions but also allows for model customization in ways that align with real-world clinical expectations. This methodological contribution has broader implications for the development of explainable AI tools in other areas of rare disease prediction, where interpretability is equally as crucial as accuracy.

Despite these advances, future research should aim to validate our approach using real-world clinical datasets from multiple institutions to assess its generalizability. Further investigation into the clinical utility and decision-support integration of SHAP-enhanced models will also be essential in strengthening the case for their broader adoption.

SHAP-based feature engineering represents a promising direction for constructing transparent and effective predictive models in healthcare. Its application to appendix cancer—a rare yet challenging condition—demonstrates how explainable AI can bridge the gap between data-driven models and clinical decision-making.

## Figures and Tables

**Table 1.** Features of the Kaggle appendix cancer prediction dataset

Category	Feature	Description
Demographic information	Age	Patient’s age in years (18-89)
	Sex	Patient’s gender (male, female, other)
	Country	Country of residence (e.g., USA, India, China)
Lifestyle factors	Smoking_Status	Smoking habit (yes/no)
	Alcohol_Consumption	Alcohol consumption level (low/moderate/high)
	Physical_Activity_Level	Physical activity level (low/moderate/high)
	Diet_Type	Type of diet followed by the patient (vegan/non-vegan)
Medical history	Family_History_Cancer	Family history of cancer (yes/no)
	Genetic_Mutations	Presence of genetic mutations (yes/no)
	Chronic_Diseases	Pre-existing chronic conditions (e.g., diabetes, hypertension)
	Radiation_Exposure	History of radiation exposure (yes/no)
Clinical measurements	BMI	Body mass index (1.1–48.1 kg/m^2^)
	Blood_Pressure	Blood pressure (90–179 mm Hg)
	Cholesterol_Level	Cholesterol level (150–299 mg/dL)
	White_Blood_Cell_Count	White blood cell count (0.5–13.7×10³/µL)
	Red_Blood_Cell_Count	Red blood cell count (2.8–7.6×10⁶/µL)
	Platelet_Count	Platelet count (150–399×10³/µL)
Diagnosis and treatment	Tumor_Markers	Tumor marker status (positive/negative)
	Symptom_Severity	Severity of symptoms (mild/moderate/severe)
	Diagnosis_Delay_Days	Days delayed before diagnosis (0–729)
	Treatment_Type	Type of treatment received (surgery, chemotherapy, radiation)
	Survival_Years_After_Diagnosis	Years survived after diagnosis (0–67.8)
Target variable	Appendix_Cancer_Prediction	Target variable: cancer prediction (yes/no)

**Table 2.** Comparison of metrics for baseline models on the original feature set

Model	Accuracy	Precision	Recall	F1-score
Random forest	0.7560	0.1506	0.1320	0.1407
XGBoost (Tuned)	0.8364	0.1310	0.0147	0.0265
LightGBM	0.8794	0.9501	0.8008	0.8691

**Table 3.** Performance metrics of LightGBM under different SHAP-based feature engineering strategies

Model	Accuracy	Precision	Recall	F1-score
Baseline (LightGBM)	0.8794	0.9501	0.8008	0.8691
Feature selection applied	0.8968	0.9893	0.8022	0.8860
Feature construction applied	0.8980	0.9915	0.8028	0.8872
Feature weighting applied	0.8986	0.9940	0.8020	0.8877

SHAP, Shapley additive explanation.

## Supplementary materials

Supplementary files are available from Harvard Dataverse: https://doi.org/10.7910/DVN/JKBPOP

Supplement 1. Python code for the machine learning methods.
